# A Long‐Lasting Skin Protectant Based on CG‐101, a Deep Eutectic Solvent Comprising Choline and Geranic Acid

**DOI:** 10.1002/gch2.202200064

**Published:** 2022-09-04

**Authors:** Marina Shevachman, Abhirup Mandal, Kevin Gelston, Samir Mitragotri, Nitin Joshi

**Affiliations:** ^1^ CAGE Bio Inc 733 Industrial Road San Carlos CA 94070 USA; ^2^ CAGE Bio Inc 181 Grand Avenue, Suite 225 Southlake TX 76092 USA; ^3^ John A. Paulson School of Engineering and Applied Sciences Harvard University Cambridge MA 02138 USA; ^4^ Wyss Institute of Biologically Inspired Engineering at Harvard University Boston MA 02115 USA

**Keywords:** alcohol‐based hand sanitizer, coronavirus, extended protection, hand hygiene, pandemic, SARS‐CoV‐2, skin protectant

## Abstract

The COVID‐19 public health crisis has spotlighted the need to improve global hygiene and sanitization. In addition to causing staggering rates of transmission and fatality, COVID‐19 has severely impacted the quality of life and mental health of global citizens. The World Health Organization (WHO) and Centers for Disease Control and Prevention (CDC) encourage hand hygiene as the first defense against the spread of infection, yet frequent handwashing is often impractical. Widely used ethanol‐based hand sanitizers provide immediate protection against pathogens on the skin, albeit short‐lived, due to their rapid evaporation. Herein, a novel, long‐lasting skin protectant formulated with biocompatible ionic liquid/deep eutectic solvents prepared using generally recognized as safe materials – choline and geranic acid (CG‐101, 5% w/w) – is described. In vitro studies demonstrated that CG‐101 inactivates bacteria and the human coronavirus, hCoV229E, for 4 h after application. Two human clinical studies demonstrate that CG‐101 does not cause skin irritation or sensitization, and a single application of CG‐101 gel imparts skin protection against microbes for significantly longer than conventional 70% ethanol‐based hand sanitizers. These data are the first to indicate that CG‐101 may be a better alternative to alcohol‐based hand sanitizers for long‐term skin protection against infectious diseases.

## Graphical abstract

The pandemic has highlighted the need to improve global hygiene. A novel skin protectant containing biocompatible the ionic liquid, choline geranate (CG‐101), provides sustained protection from harmful pathogens including human coronavirus for 4 h. Data from human trials demonstrate that CG‐101 does not cause skin irritation, and a single application of CG‐101 gel imparts protection significantly longer than ethanol‐based sanitizers.

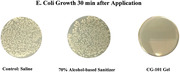

## 1 Introduction

The zoonotic virus, severe acute respiratory syndrome coronavirus 2 (SARS‐CoV‐2), is responsible for the coronavirus disease (COVID‐19) associated with severe respiratory distress, fever or chills, cough, sore throat, congestion, nausea, and diarrhea.^[^ ^1^ ^]^ The World Health Organization (WHO) officially declared COVID‐19 as a global pandemic on March 11, 2020.^[^ ^2^ ^]^ Since then, the Centers for Disease Control and Prevention (CDC) has reported that COVID‐19 has infected > 74 million people in the United States alone, with 880 000 deaths as of January 29, 2022. Unfortunately, older adults and individuals with underlying health conditions‐ such as heart disease, lung disease, autoimmune disorders, and diabetes‐ are at a higher risk of mortality from viral infection.^[^ ^3^, ^4^ ^]^ Since the start of the pandemic, the number of confirmed cases, deaths, and economic losses arising from COVID‐19 have amassed to an alarming level.^[^ ^5^ ^]^

As it stands, the severity of the pandemic highlights the need for novel, prophylactic scientific advancements to minimize the initial transmission of pathogens. Indeed, harmful pathogens can be highly contagious and can be easily transmitted even when the infected subjects are asymptomatic, presymptomatic, or have mild symptoms.^[^ ^6^ ^]^ While antiseptics, antibiotics, vaccines, and antiviral drug discovery remain paramount to addressing these global threats, slowing pathogen transmission can be attainable with relatively simple behavioral changes from each individual.^[^ ^7^, ^8^ ^]^ Regulatory agencies, including the CDC and WHO, have issued guidance for preventive actions, including hand‐washing for 20 s, donning masks responsibly, and 6 feet of social distancing.^[^ ^9^, ^10^ ^]^ While these safety measures are simple in principle, strict compliance with these requirements can be challenging to sustain over long periods. Since it is neither practical nor viable to meet these requirements all the time, improving compliance will significantly affect the outcome.

Since 1994, the US Food and Drug Administration has proposed ethanol (60–95% v/v) as safe and effective for hand sanitizers.^[^ ^11^ ^]^ As such, WHO recommends using formulations containing 80% v/v ethyl alcohol or 75% v/v isopropyl alcohol to effectively inactivate pathogens, including acute respiratory syndrome coronavirus (SARS‐CoV), Middle East respiratory syndrome coronavirus (MERS‐CoV), and most recently, SARS‐CoV‐2 (COVID‐19).^[^ ^12^ ^]^ However, the biggest shortcoming of such alcohol‐based formulations is that their protective properties only last until the alcohol evaporates, that is, a time frame of 15 s to 2 min. Once the narrow window of protection is over, the users’ hands are again vulnerable to infection and further transmission. Hence, developing an effective hand sanitizer that can generate long‐lasting protective effects will offer significant benefits for minimizing pathogen transmissions and maximizing virus inactivation in a pandemic situation like the COVID‐19 outbreak.^[^ ^11^, ^13^, ^14^, ^15^ ^]^

There has been no significant innovation in hand sanitizers since the 1960s when alcohol‐based sanitizers were first introduced.^[^ ^16^ ^]^ Here, we report a skin protectant composed of CG‐101, an ionic liquid (IL)/deep eutectic solvent (DES), comprising choline – an essential nutrient, and geranate – an unsaturated fatty acid ion, formulated in ethanol/water gel. CG‐101 (CAGE) was first characterized and disclosed by Zakrewsky and colleagues.^[^ ^17^ ^]^ The novel skin protectant formulation exploits the unique properties of ionic liquids and deep eutectic solvents (negligible volatility, strong antimicrobial activity, biocompatibility) to enable a long‐lasting skin protectant compared to conventional alcohol‐based sanitizers that provide only temporary protection.

Over the past decade, ILs/DES such as CG‐101 has emerged as a safer alternative to conventional organic solvents, owing to their low viscosity, thermal stability, low corrosivity, and non‐flammability under ambient conditions.^[^ ^18^, ^19^ ^]^ Biocompatible‐ionic liquids (B‐ILs) are unique in their environmental compatibility. All choline‐based B‐ILs have demonstrated biodegradability and low toxicity by the technical requirements set by the European Standards method.^[^ ^20^ ^]^ Recently, much excitement has surrounded the possible uses for antimicrobial, cytotoxic, and permeable B‐IL formulations in biomedical applications, including the use of CAGE for antiseptic agents and in drug delivery.^[^ ^17^, ^21^ ^]^

In this relevant and timely research study, we demonstrate the safety and efficacy of the B‐IL‐based long‐lasting skin protectant in providing long‐term skin protection against pathogens, including human coronaviruses (hCoV229E). CG‐101 has already demonstrated broad‐spectrum antimicrobial properties against 47 American‐type culture collection (ATCC) strains of bacteria, viruses, and fungi.^[^ ^17^, ^22^, ^23^, ^24^ ^]^ Since antimicrobial action is mediated through the general mechanism of lipid extraction and membrane disruption,^[^ ^22^ ^]^ we anticipate that these properties may support its activity against coronaviruses with a lipid envelope. Ultimately, a long‐lasting skin protectant that can effectively inactivate coronaviruses and other pathogens over several hours post‐application will provide people the freedom to carry on without constant concern over their health and safety.

## 2 Results

### 2.1 Characterization

**Figure 1:**
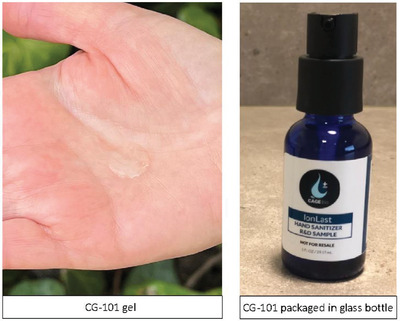
CG‐101 skin protectant gel. Images of 5% w/w CG‐101 gel on the skin and packaged in a glass bottle with a plastic pump dispenser for long‐term storage. Picture of glass bottle published with permission from CAGE Bio Inc.

CG‐101 gel is a proprietary formulation containing 5% w/w CG‐101, ethanol, and water as solvents, glycerin, a gelling agent, aloe vera, and a small amount of a fragrance material. The novel CG‐101 formulation is a viscous, colorless‐to‐light‐yellow gel with a light citrus fragrance (**Figure** **1**). It has a viscosity of ≈1500 cP at room temperature conditions, making it easy to dispense and spread on hands (Figure 1).

The stability of CG‐101 gel was evaluated at room temperature and accelerated storage conditions (40 °C). Chemical stability was evaluated for up to 3 months, whereas physical and microbiological stability was assessed through 12 months. Notably, the pH of CG‐101 gel remained close to neutral at ≈7.7, and the viscosity of the gel remained within acceptable limits of 1,000–1,500 cP throughout 12 months. Microbial limit testing was also performed considering that CG‐101 gel contains a large amount of water; there was no microbial growth in the gel throughout 12 months of storage. Therefore, CG‐101 packaged in glass bottles meets all quality control parameters required for 1, 3, 6, and 12 months of stable storage at room temperature and 40 °C (Table S1, Supporting Information).

### 2.2 CG‐101 Demonstrates Bactericidal Activity

**Figure 2:**
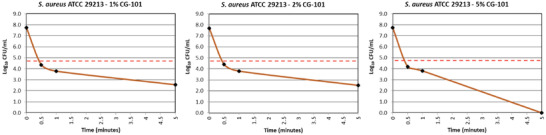
Short time‐course analysis of CG‐101 bactericidal activity. Time‐course regression graphs demonstrating Log_10_ reduction in *S. aureus* CFU mL^−1^ after 0.5, 1‐, and 5‐min exposure to CG‐101 (1%, 2%, 5%). Dotted red line indicates > 3 Log_10_ reductions from the initial inoculum (*n* = 3; two replicates/experiment).

A time‐kill study confirmed the bactericidal efficiency of varying concentrations of CG‐101 against *Staphylococcus aureus (S. aureus)* (ATCC 29213). Culture plates were treated with aqueous solutions of CG‐101 (1%, 2%, 5%) and subsequently inoculated with *S. aureus* for 0.5, 1, and 5 min before CG‐101 neutralization. The plates were quantitatively assessed for Log_10_ and percent reduction in *S. aureus* colony‐forming units (CFU) per volume compared to the initial inoculum. For all concentrations of CG‐101, the Log_10_ reduction in CFU mL^−1^ was > 3.00 at 0.5 and 1 min, and > 5.00 by 5 min (**Figure** **2**). In fact, the Log_10_ reduction in *S. aureus* CFU mL^−1^ was ≥ 7.73 after 5 min in CG‐101 5% (Figure 2). Furthermore, the percent reduction in CFU mL^−1^ was > 99.9% for all timepoints at all concentrations (Table S2, Supporting Information). In other words, 99.9% of *S. aureus* colonies were killed within 30 s of exposure to CG‐101 aqueous solutions. Considering that CG‐101 5% showed the most effective antimicrobial activity, 5% CG‐101 gel, a formulation containing 5% CG‐101, is anticipated to perform equally well.

### 2.3 CG‐101 Deactivates Human Coronavirus hCoV229E

**Figure 3:**
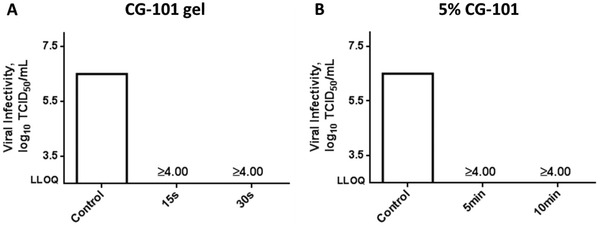
Virucidal efficacy test against human coronavirus strain 229E (hCoV229E). Quantification of coronavirus strain 229E viral infectivity of MRC‐5 cells after A) 15 and 30 s treatment with 5% CG‐101 gel (contains 5% w/w CG‐101) and B) 5 and 10 min treatment with CG‐101 aqueous solution (5% w/w), in comparison to untreated control. Log_10_ reductions in viral infectivity are included above the 15 and 30 s time‐point bars; viral infectivity (measured by viral titers with fibroblast cytotoxicity) is displayed as TCID_50 _mL^−1^ values; (*n* = 4). LLOQ, the lower limit of quantification; TCID_50 _mL^−1^, 50% tissue culture infectious dose.

Based on the previously demonstrated broad‐spectrum antimicrobial properties of CG‐101,^[^ ^23^ ^]^ we anticipated that CG‐101 and the gel formulation containing 5% w/w CG‐101 would effectively deactivate coronaviruses through disruption of the viral envelope containing a glycoprotein and phospholipid bilayer. We evaluated the anti‐virucidal properties of the 5% CG‐101 gel formulation and CG‐101 aqueous solution (CG‐101, 5% w/w in purified water) against human coronavirus strain 229E (hCoV229E). To do this, we infected human fibroblasts (MRC‐5) with hCoV229E and performed a virucidal suspension test (in‐vitro, time‐kill method) based on industry/regulatory‐relevant global standardized methodologies (ASTM E1052‐20). There was > 4.00‐fold Log_10_ reduction in the hCoV229E viral infectivity of MRC‐5 cells following 15 and 30 s exposure to 5% CG‐101 gel compared to untreated control (**Figure** **3**, Table S3, Supporting Information). In fact, the percent reduction for both 15 and 30 s time points was > 99.99% (Table S3, Supporting Information). Similarly, we evaluated the virucidal activity of CG‐101 aqueous solution (5% w/w) using the quantitative suspension test for 5 and 10 min exposure times. The Log_10_ reductions and percent decrease in viral infectivity from the initial population of hCoV229E were also found to be > 4.00% and > 99.99%, respectively (Figure 3, Table S4, Supporting Information).

### 2.4 Long‐Lasting Bactericidal Efficacy of 5% CG‐101 Gel

**Figure 4:**
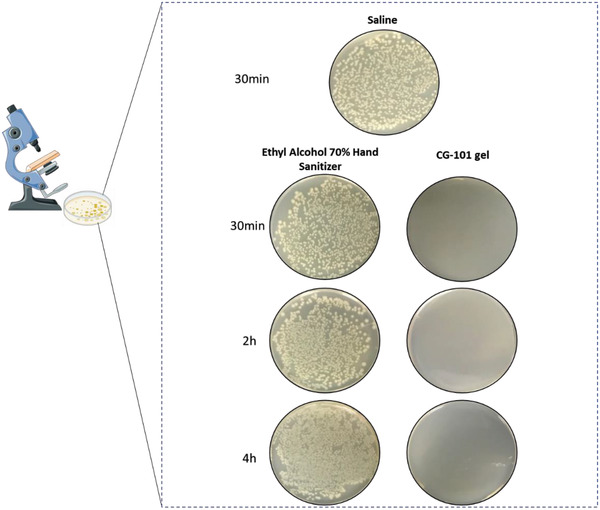
Long‐lasting in‐vitro antibacterial efficacy against *E. coli*. Qualitative assessments of colony growth on plates inoculated with *E. Coli* at 30 min, 2 h, or 4 h post‐treatment with saline (negative control), 70% ethyl alcohol hand sanitizer (comparator), and 5% CG‐101 gel (test). (*n* = 2, two replicates/experiment for 30 min and 2 h and *n* = 3 replicates for 4 h of post‐application).

To determine the prolonged protective effects of incorporating 5% CG‐101 in a gel formulation, we conducted an in vitro efficacy study using *Escherichia coli* (*E. coli*) (following modified American Society for Testing and Materials (ASTM) E1153 methodologies). A single, 1 mL application of the test article (5% CG‐101 gel), comparator (70% ethyl alcohol hand sanitizer), and negative control (saline) products were applied to sterilized glass surfaces and left for three periods of time: 30 min, 2 h, and 4 h. After the antiseptic treatment and respective wait periods, the glass slides were inoculated with 10 µL of *E. coli* suspension for a total exposure time of 5 min. The test article was then chemically neutralized, and all samples were transferred to agar plates for 48 h at 37 °C to assay colony growth visually. The test article containing 5% CG‐101 effectively prohibited all *E. coli* growth 30 min, 2 h, and 4 h after initial application, indicating persistent antimicrobial activity (**Figure** **4**). In contrast, *E. coli* colony growth was observed in increasing amounts 30 min, 2 h, and 4 h after the initial application of 70% ethyl alcohol hand sanitizer (Figure 4). In fact, the colony growth seen 30 min after treatment with 70% ethyl alcohol was indistinguishable from the growth observed in the saline negative‐control plate for the same timepoint (Figure 4). This indicates that conventional alcohol‐based hand sanitizers have limited ability to provide long‐lasting protective effects against microbes. On the contrary, the incorporation of 5% CG‐101 in the formulation provided a clear indication of durable protection against pathogens, thus potentially reducing disease transmission during a pandemic.

### 2.5 Safety of CG‐101 in Human Volunteers

The safety of CG‐101 (100% concentration) was tested in a 3‐week human repeat insult patch test (HRIPT) in 52 adult human volunteers (NCT04498676). The 100% concentration of CG‐101 used in this study was 20 times higher than that used in 5% CG‐101 gel. For this study, CG‐101 was applied to the subjects’ back over a marked 2 × 2 cm^2^ area and covered with an occlusive hypoallergenic patch to maximize penetration; distilled water was used as a negative control. The subjects removed the patches on their own after 24 h. At the next site visit, the study investigator observed the application area for reaction on a 5‐point scale (0–4) and applied a new patch on subjects without reaction. The subjects reported to the site for a total of nine visits, that is, three times a week for 3 weeks. No subjects showed any skin reactions in the first three visits (week 1). Between visits 4 through 9 (weeks 2 and 3), eight subjects experienced some skin irritation; however, no follow‐up treatment was required. The other forty‐three subjects did not display any irritation throughout the 21‐day study (weeks 1–3). There were no other adverse events reported in the study. Furthermore, upon completion of the 21‐day study period and a rest period of two weeks, 100% CG‐101 was applied with occlusion to a randomly assigned unexposed area and did not cause a sensitization response after 24 and 48 h.

### 2.6 Prolonged Residual Antimicrobial Efficacy in Humans

A clinical study was performed to test the effectiveness of 5% CG‐101 gel in providing extended protection against microbes. This GLP clinical study (NCT04495920) was approved by an IRB and conducted in compliance with reasonable clinical practice regulations. 5% CG‐101 skin protectant gel was applied to randomly assigned test sites on the forearms of healthy human volunteers. The test sites were then challenged by applying *S. aureus* to the area at various times after applying the gel. The residual antimicrobial efficacy was tested using a modification of the standardized test method described in ASTM E2752‐10 (2015) in 12 healthy subjects. A significant (≥5.15) Log_10_ reduction in *S. aureus* was observed at 30 min, 2 h, and 4 h post‐ 5% CG‐101 gel application.

**Figure 5:**
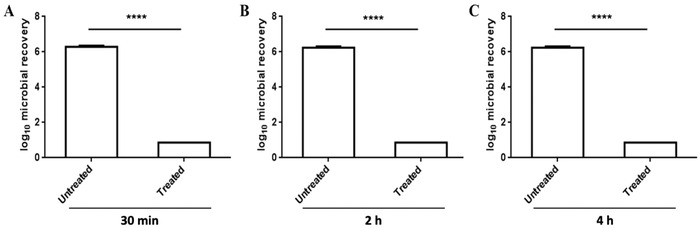
Residual antimicrobial efficacy against *S. aureus* in humans. The means of Log_10_ microbial recovery in comparison to untreated subjects A) immediately (30 min), B) 2 h, and C) 4 h following application of the test product: 5% CG‐101 gel. Data are averages ± SEM (standard error of the mean), statistics by two‐way ANOVA with Bonferroni's multiple comparison test. ****p < 0.0001 (*n* = 11).

After 30 min of air‐drying post gel application, the mean Log_10_ microbial recovery for the treated skin site was 0.86 ± 0.00 (Note that 0.86 Log_10_ CFU cm^−2^ is the lowest detectable limit of the method) in contrast to 6.30 ± 0.05 for the untreated skin site (**Figure** **5**). Furthermore, the Log_10_ microbial recovery, 2 h following the 5% CG‐101 gel application, was found to be 0.86 ± 0.00 compared to 6.24 ± 0.07 for the untreated skin sites (Figure 5). Interestingly, even after 4 h post‐application, the Log_10_ microbial recovery for the treated skin site was also 0.86 ± 0.00 in comparison to 6.24 ± 0.12 for untreated skin (Figure 5). The 5% CG‐101 gel thus demonstrated prolonged microbial protection for up to 4 h after a single application (Table S5, Supporting Information).

## 3 Discussion

SAR‐CoV‐2, the virus that causes COVID‐19, has evolved into a global pandemic that has infected > 74 million people in the United States since March 2020. While the long‐term development of effective vaccines and antiviral therapies remains at the forefront of global efforts, a more immediate response plan should involve strategies that break the chain of initial viral transmission.^[^ ^25^ ^]^ Current evidence suggests that SARS‐CoV‐2 predominantly spreads from person to person via saliva and respiratory secretions or droplets.^[^ ^26^ ^]^ Other modes of transmission may include contaminated objects or surfaces (fomite transmission), fecal‐oral, bloodborne, mother‐to‐child, and animal‐to‐human transmission.^[^ ^27^ ^]^ The relative infectivity of various routes is unclear and requires additional epidemiological data and mechanistic studies of virus entry. For example, angiotensin‐converting enzyme 2 (ACE2), the cell receptor for SARS‐CoV‐2 entry, is abundantly present in blood vessels/capillaries of the skin, the basal layer of the epidermis, and hair follicles.^[^ ^28^, ^29^ ^]^

Hand hygiene is widely recognized for playing a significant role in limiting the spread and transmission of infectious diseases.^[^ ^14^ ^]^ To cope with the COVID‐19 pandemic, the CDC has explicitly recommended at least 20‐s of handwashing with soap and water whenever possible. Furthermore, hand washing can be burdensome, disruptive, and simply impractical in many environments.^[^ ^30^ ^]^ A common alternative to hand washing is the persistent use of alcohol‐based hand sanitizers, with at least 60% alcohol. Unfortunately, the protective effect of alcohol lasts only until the alcohol evaporates, that is, less than a couple of minutes. Frequent handwashing with soap and water and repeated application of evaporative alcohol‐based sanitizers may even dry out and damage the skin, potentially leading to dermatitis.^[^ ^31^ ^]^

Inspired by the fact that CG‐101 exhibits broad‐spectrum antimicrobial properties against a range of bacteria, viruses, and fungi, we developed an alcohol‐based skin protectant containing 5% CG‐101 as a countermeasure to COVID‐19 infection and transmission. CG‐101 inactivates pathogens by extracting lipids and thus disrupting the cell membrane or viral envelope.^[^ ^22^ ^]^ CG‐101 selectivity toward pathogens relative to mammalian cells stems from the higher content of phosphatidylcholine in mammalian cells. Previous studies have provided compelling evidence on the ability of CG‐101 against drug‐resistant pathogens. In addition, CG‐101 was shown to be capable of rapidly eradicating mature biofilms of a suit of clinically relevant ESKAPE pathogens, even as dilute formulations. CG‐101 disrupts the extracellular polymeric substance layer of biofilms and destroys the underlying cells upon contact. The potency and time required for full biofilm eradication for CG‐101 compare favorably to literature values reported for other common standard‐of‐care topical antiseptics. Established methicillin‐resistant strains, as well as those demonstrating resistance to imidazolium‐based ionic liquids, were both shown to be effectively eradicated by CG‐101, indicating that even the risk of cross‐resistance by existing resistance mechanisms is extremely low for CG‐101. More importantly, unlike ethanol, which is highly volatile, CG‐101, being an ionic liquid/deep eutectic solvent, has a very low vapor pressure and can remain on the skin long after ethanol evaporates. The long‐lasting presence of CG‐101 on the skin is expected to offer enduring protection against infectious pathogens, including coronaviruses, even with less frequent hand sanitization.

Human volunteer studies confirmed the safety of the active CG‐101 compound (≈100% concentration). We adopted the HRIPT protocol, an industry‐standard test for evaluating the potential of a test material to induce sensitization in humans after repeated exposure.^[^ ^32^ ^]^ Eighty percent of adult human volunteers treated with a 20x higher concentration of CG‐101 than that used in 5% CG‐101 gel did not display any irritation throughout the 21‐day study. Following completion of the study and a rest period of two weeks, CG‐101 application to a different part of the body also did not create any sensitization response. These data indicate that the bio‐ionic liquid/deep eutectic solvent, CG‐101, is a safe additive for topical products. Therefore, it was also prudent to investigate how effective lower doses of CG‐101 are as antimicrobial agents. A short‐term, time‐kill bacterial study confirmed that lower doses of CG‐101(1%, 2%, and 5%) are indeed effective at eradicating 99.9% of *S. aureus* colonies within 30 s (Figure 2). Together, these results corroborated the high potential for CG‐101 as the active ingredient in a gentle yet antimicrobial and bactericidal skin protectant.

Considering the pressing epidemiological need for a long‐lasting skin protectant, specifically, an in‐vitro bactericidal assay was conducted to compare the longevity of the antibacterial efficacy of 5% CG‐101 gel in contrast to currently marketed alcohol‐based products. There was a striking reduction in the number of test microorganisms that could survive on surfaces even 30 min, 2 h, and 4 h after application of 5% CG‐101 gel compared to conventional alcohol‐based products. In fact, it is concerning to report that there was no notable difference in the microbial growth on surfaces just 30 min after treatment with alcohol‐based hand sanitizers as compared to saline, the negative control (Figure 4); this indicates the short duration of protection rendered by ethanol. Fortunately, the protective effects of 5% CG‐101 gel against *E. coli* (and possibly other pathogens) persisted for up to 4 h. These critical results highlight the impact that novel biocompatible‐ionic liquids/deep eutectic solvents, such as CG‐101, could have on halting pathogenic transmission caused by poor hand hygiene.

The encouraging results from the bactericidal efficacy studies motivated us to test 5% CG‐101 gel against human coronaviruses. As expected, 5% CG‐101 gel and CG‐101 (5% w/w aqueous solution) demonstrated excellent virucidal efficacy against hCoV229E. We report that 5% CG‐101 gel yielded > 4.00 Log_10_ reductions in viral titers following 15 and 30 s exposure. Moreover, CG‐101 (5% w/w aqueous solution) generated similar Log_10_ reductions (>4.00) from the initial viral population after 5 and 10 min exposure times (Figure 3). Importantly, these data are the first to indicate that human coronaviruses may be highly susceptible to CG‐101 and its novel formulation, 5% CG‐101 gel.

A residual antimicrobial efficacy study was undertaken to measure the relative persistence of antibacterial activity under controlled clinical test conditions. The results demonstrated a significant (≥5.15) Log_10_ reduction in *S. aureus* on subjects’ skin 30 min, 2 h, and 4 h after the initial application (Figure 5). These clinical results further support the efficacy of 5% CG‐101 gel for persistent pathogen inactivation. Considering these promising clinical and in vitro data, we investigated the long‐term stability of 5% CG‐101 gel packaged in glass bottles for up to 12 months. The stability testing demonstrated no deterioration of principal characteristics, including appearance, CG‐101 and ethanol assay, pH, viscosity, impurities, or microbial contamination (Table S1, Supporting Information). 5% CG‐101 gel is therefore safe to package and store for up to 1 year, which implies its potential for global impact. However, further clinical studies are warranted to demonstrate the prolonged protection of CG‐101 against infectious pathogens on human skin. Until then, the long‐lasting antimicrobial protection of CG‐101 serves as a potentially promising complement to the existing CDC/WHO guidelines for good hand hygiene.

## 4 Experimental Section

### Manufacturing of CG‐101 and CG‐101 Gel

To begin the manufacturing process, the ionic liquid CG‐101 was formed by gradually adding a choline bicarbonate solution to geranic acid at room temperature and gently mixed for 8 h. Completion of the reaction was confirmed by measuring pH and water content. The resulting CG‐101 ionic liquid was sequentially mixed with the required amounts of glycerin, fragrance, and ethanol. A cellulose gelling agent was then dispersed in this solution and mixed in a high shear mixer until a homogeneous gel was obtained. Finally, aloe vera dissolved in water was added to the CG‐101 gel. The obtained CG‐101 gel was packaged in amber glass bottles with a plastic pump dispenser (Figure 1).

### Assay and Impurities

Assay and impurities of CG‐101 were measured by quantifying the amount of choline and geranic acid in the sample using reversed‐phase HPLC gradient methods.

### Viscosity

The viscosity of CG‐101 and CG‐101 gel was measured using a Brookfield LVT viscometer with spindle #4 at 6 rpm.

### Clinical Safety Study of CG‐101

The skin irritation and sensitization potential of CG‐101 were evaluated in a 21‐day human repeat insult patch test (HRIPT) in 52 volunteers. This cosmetic study was approved by an IRB and conducted by AMA Laboratories Inc. in New York. CG‐101 (100%, liquid) was applied on the back of volunteers over a 2 × 2 cm^2^ area on each of nine visits over 3 weeks (21 days). The application area was occluded with hypoallergenic tape. The subjects were required to keep the tape for 24 h. The application site was evaluated for skin irritation on a 5‐point scale (0–4) at each visit. Any subjects that showed irritation rated at 3 or higher were withdrawn from the study and monitored for changes. After completion of the 3‐week test period, the subjects were given a 10 to 14‐day rest, after which CG‐101 solution was applied to a previously unexposed site. The subjects were then evaluated for any skin reactions after 24 and 48 h. Disclaimer: Informed signed consent was obtained from all patients or next of kin.

### Bactericidal Time‐Kill Study

As a first step in performing the time‐kill assay, *Staphylococcus aureus* (*S. aureus*) ATCC 29213 was tested in accordance with the neutralization evaluation procedure utilizing 1%, 2%, 5%, and 10% concentrations of CG‐101, recovery on semi‐solid medium at T‐0 (≈10 seconds) and T‐5 min (ASTM E1054‐08) and colony counts.  Following acceptable neutralization results, the time‐kill assay was performed using 1%, 2%, and 5% concentrations of CG‐101 and colony counts were determined following exposure of CG‐101 after 30 s, 1 min, and 5 min.  The neutralization and time‐kill testing were performed in triplicate, and all colony counts were performed in duplicate. Final counts were converted to Log_10_. The neutralization effectiveness tube (Test A), neutralization toxicity tube (Test B), and CG‐101 tubes (Test D) were compared to the organism viability tube (Test C) using a Student's *t*‐test (α = 0.05). Neutralization was considered adequate when Test A was not significantly different from Test C (*p* > 0.05) and Test D was significantly smaller than Test C (*p* > 0.05). The neutralization medium was considered non‐toxic if Test B was not significantly different from Test C (*p* > 0.05).

### In Vitro Bactericidal Efficacy

To determine the relative bactericidal efficacy of the 5% CG‐101 gel in contrast to a conventional alcohol‐based product, ASTM E1153, the standard test method for the efficacy of sanitizers recommended for inanimate non‐food contact surfaces was modified and employed accordingly. Briefly, pre‐cleaned surfaces were transferred into sterile Petri plates using sterile forceps, covered with 1 mL of the test substances, and held for 30 min at ambient temperature to dry. The inoculation with 10 µL of *E. coli* suspension in a 3.2 × 10^8^ CFU mL^−1^ concentration was performed at 30 min, 2 h, and 4 h. After each inoculation, the exposure time was 5 min. Samples at 30 min and 2 h were tested in duplicate while the samples at 4 h were tested in triplicate. After 5 min, the test compound was neutralized, and the viable bacteria were resuspended. A neutralization study was conducted to assure that the neutralizers used for the study quenched the antimicrobial activity of each test material and were not toxic to the challenge species. After neutralization, the samples were plated on agar plates and transferred into the incubator set at 37 °C for 48 h. An average of at least ≥ 104 CFU of bacteria were recovered from the negative and neutralization controls. The number of viable organisms on the agar plates treated with 5% CG‐101 gel was then compared visually to the negative control (untreated) and the comparator (70% ethyl alcohol hand sanitizer) following 48 h incubation.

### Virucidal Suspension Test

The virucidal test was based upon ASTM E1052‐20, standard practice to assess the activity of microbicides against viruses in suspension. All testing was performed per Good Laboratory Practices (GLP), as specified in 21 CFR Part 58 at BioScience Laboratories, Inc. (BSL), Bozeman, Montana. The characterization of the identity, strength, purity, composition, stability, and solubility of the test product(s) was performed by CAGE Bio, Inc. The percent and Log_10_ reductions from the initial viral strain(s) population were determined following exposure to the test product, 5% CG‐101 gel, for 15 and 30 s, and to the test product, CG‐101 (5% w/w), for 5 and 10 min. Testing was performed in one replicate, and plating was conducted in four replicates. Coronavirus (alphacoronavirus) strain 229E (ATCC #VR‐740) and MRC‐5 (ATCC #CCL‐171; human lung fibroblast cells) were used for the study.

### Residual Antimicrobial Efficacy Test

The residual antimicrobial efficacy testing was carried out using a modification of the standardized test method described in ASTM E2752‐10 (2015), the standard guide for evaluating the residual effectiveness of antibacterial personal cleansing products. The study was conducted in compliance with Good Clinical Practice Regulations, Good Laboratory Practice Regulations, the standard operating procedures of BioScience Laboratories, Inc., the study protocol, and any protocol amendments. The study plan and documents, including the protocol and informed consent, were approved by an Independent Review Board (IRB). Bacterial recoveries were assayed after applying test material, using the forearms as a substrate. Twelve test subjects (aged 18–65 years) with healthy skin were used in this study, and the test product was applied on one arm and had the other arm untreated as the negative control. The test sites on both forearms were inoculated with suspensions containing *S. aureus* (ATCC #6538), immediately after the 30‐min product drying time and at approximately 2 and 4 h following test material applications. The test sites were sampled using the cup scrub procedure approximately 20 min following each inoculation. The Log_10_ microbial recoveries of treated versus untreated sites were the basis for assessing the residual antimicrobial effectiveness of the test product.

A neutralization study was also performed to assure that the neutralizers used in the recovery medium quench the antimicrobial activity of each test material and were not toxic to the challenge species. Study procedures were based on ASTM E 1054‐08(2013), standard test methods for evaluation of inactivators of antimicrobial agents. *S. aureus* (ATCC #6538) was used as the challenge species in the neutralization study. Disclaimer: Informed signed consent was obtained from all patients or next of kin.

Tables S6–S8, Supporting Information present statistical summaries of treated and untreated mean Log_10_ microbial recoveries of *S. aureus* (ATCC #6538) for the immediate (30 min), 2‐h, and 4‐h time‐points, and mean Log_10_ microbial differences from untreated controls.

The initial and final populations of the *S. aureus* (ATCC #6538) inoculum suspension used in testing are presented in Table S9, Supporting Information.

### Statistical Analysis

Results are depicted as average ± SEM. Parametric data were analyzed by two‐way ANOVA with Bonferroni's multiple comparison test. The Quantal test (Spearman–Kärber Method) was applied to calculate virus titer for the virucidal suspension test. No control of bias was performed. The MiniTab® Version 18 statistical computer package was used for all statistical calculations for the antimicrobial efficacy test. A blocked, one‐factor analysis of variance (ANOVA) model was used:

(1) $$\begin{array}{c} y=\text{Blocks}+\text{Sample}\;\text{Time}+e \end{array}$$

where:

*y* = Log_10_ Reduction = Untreated Log_10_ Recovery − Treated Log_10_ Recovery;

Blocks = One subject will use both configurations;

Sample Time = 1 (if immediate), 2 (if 2 h), 3 (if 4 h);

*e* = Error Term.

Detailed statistical analysis are presented in Tables S5–S13 and Figures S1 and S2, Supporting Information.

## Conflict of Interest

CAGE Bio has a license to and ownership of patents pertaining to ionic liquids. A.M., M.S., K.G., and N.J. are employees and shareholders of CAGE Bio. S.M. is a shareholder and board member/consultant to Liquideon, CAGE Bio, and i2O Therapeutics.

## Author Contributions

M.S. and K.G. performed characterization and formulation development activities; M.S., A.M., and N.J. designed the GLP antibacterial and virucidal studies and data analysis; M.S. and N.J. designed the clinical safety study and data analysis; A.M. performed data summary, A.M. and S.M. summarized findings and wrote the manuscript with the help of all authors.

## Supporting information

Supporting Information

## Data Availability

The data that support the findings of this study are available in the supplementary material of this article.
