# Supplementary material for: A Long‐Lasting Skin Protectant Based on CG‐101, a Deep Eutectic Solvent Comprising Choline and Geranic Acid
Source: Glob Chall. 2022 Sep 4;6(11):2200064. doi: 10.1002/gch2.202200064 (PMC9638410; doi:10.1002/gch2.202200064)

Global Challenges
Open Access

Supporting Information

for *Global Challenges,* DOI: 10.1002/gch2.202200064

A Long-Lasting Skin Protectant Based on CG-101, a Deep Eutectic Solvent Comprising Choline and Geranic Acid

*Marina Shevachman, Abhirup Mandal, Kevin Gelston, Samir Mitragotri, and Nitin Joshi**

Supplementary Materials

Materials and Methods

1. Virucidal suspension test. MRC-5 cells were maintained as monolayers in disposable cell culture labware in accordance with protocol. Prior to testing, host cell cultures were seeded onto multi-well cell culture treated plates. Cell monolayers reached 80% to 90% confluency and were less than 48h old before inoculation with the virus. Briefly, a 0.5 mL aliquot of test virus(s) was used for the virucidal suspension test. The virus was added to a vial containing 4.5 mL of the test product(s). The test virus(s) was exposed to the 5% CG-101 gel for 15s and 30s and to the CG-101 aqueous solution (5% w/w) for 5min and 10min, timed using a calibrated minute/second timer. The calibrated minute/second timer was started within ± 1s of adding the challenge suspension. Immediately after exposure(s), the test virus(s)/product suspensions were neutralized in appropriate neutralizer, mixed thoroughly, and serially diluted in maintenance medium (MM). Each dilution was plated in four replicates. All other controls were prepared following the standard protocol. Viral and toxicity titers are expressed as -Log_{10} of the 50% titration endpoint for infectivity. To calculate the viral titer, a 50% tissue culture infectious dose ($TCID_{50}$) calculation – the Quantal test (Spearman-Kärber Method) – was applied.

$$\text{Log } TCID_{50} = L - d\,(s - 0.5)$$

Where:

L = -$\log_{10}$ of the lowest dilution;

d = difference between dilution steps;

s = sum of proportions of positive wells.

The Log_{10} of infectivity reduction was calculated as follows:

Log_{10} Reduction = ($\log_{10}$ $TCID_{50}$ of the Virus Control) - ($\log_{10}$ $TCID_{50}$ of the Virucidal Suspension Test)

The percent reduction was calculated as follows:

$$\%Reduction = \left(1 - \frac{TCID50test}{TCID50\ virus\ control}\right)X100$$

2. Residual antimicrobial efficacy test period. Each subject had one forearm assigned to the test product and the other forearm remained as an Untreated Control. The sites on each forearm were randomized for the sample times, 30min, 2h and 4h post product application. Subjects performed a 30s wash with 5 mL of nonmedicated soap and a 30s rinse to remove any dirt or oil from the forearms prior to the test. Subjects dried their hands and forearms with disposable paper towels. The temperature of the water was controlled at 40 °C ± 2 °C. The forearms were decontaminated with 10 mL of 70% Ethyl alcohol (EtOH) dispensed over the surface of both forearms and allowed to air-dry. After completion of the forearm disinfection, each subject waited at least 5 min prior to continuing to the next step. The subjects were instructed to not touch anything with their forearms during this time. Using a surgical skin marker, 2" x 6" test sites were marked out on the volar

surface of each forearm. Three areas approximately 2 cm in diameter on the skin of the volar surface of each forearm were marked. The application test sites were re-marked as needed.

2.a. Test product application. A single 3-mL aliquot of test product was dispensed onto the subjects' forearm. A technician wearing sterile gloves evenly applied the test product over the entire 2" x 6" test site. The inoculation process began 30min ± 3min after the test product was applied to the test site.

2.b. Inoculum application. Inoculum was prepared by following the study protocol. At three-time points following the 30-minute air-dry after test product application, a 10 µL (0.01 mL) aliquot of the challenge inoculum was applied to the randomly assigned test sites on each forearm, and a heat-polished glass rod was used to distribute the inoculum over the demarcated - 2cm diameter test area (but not reaching the edges of the demarcated area) and allowed to air-dry for at least 20min. Inoculations began immediately after the 30min ± 3min drying time wait, 2h ± 15min, and 4h± 15minafter the completion of the test product application. The contaminated sites on each forearm were sampled using the Cup Scrub Technique following the 20min inoculum air dry.

2.c. Cup scrub technique. A sterile stainless-steel cylinder with an inside area of 3.46 cm^2 was held firmly onto a site to be sampled. A 1.0 mL aliquot of Sterile Stripping Suspending Fluid with product neutralizers (SSF++) was dispensed into the cylinder, and the skin area inside the cylinder was massaged in a circumferential manner for 1min ± 10s with a heat-polished glass rod. The 1.0 mL of SSF++ was removed with a pipette and transferred to a sterile test tube. A second 1.0 mL aliquot of SSF++ was dispensed into the cylinder, and the skin area was massaged for 30s ± 5s with a heat-polished glass rod. The second 1.0 mL aliquot was then pooled in the test tube with the first aliquot. Gauze soaked in 70% Ethyl alcohol (EtOH) was used to decontaminate the sites.

2.d. Plating and data collection. Aliquots of the microorganism suspension (10° dilution) was serially diluted in Butterfield's Phosphate Buffer Solution with product neutralizers (BBP++), as appropriate. Duplicate spread plates were prepared from appropriate dilutions using Mannitol Salt Agar (MSA) and incubated at 35 °C ± 2 °C for approximately 48h or until sufficient growth is observed. *S. aureus* (ATCC #6538) produced golden-yellow colonies on MSA, and only those colonies were counted. If colonies on one of the plates were uncountable, the count from the remaining plate was used. Average colony counts of 0 at the -1 dilution was recorded as <5.00 CFU/mL.

2.e. Microbial recoveries and Statistical analysis. The MiniTab® Version 18 statistical computer package was used for all statistical calculations. The estimated $\log_{10}$ number of viable microorganisms per cm^2 recovered from each sample site were designated the “R-value.” To convert the volumetric measure of the sample into the number of colony-forming units per square centimeter (cm^2), the following formula was employed:

$$R = Log_{10}\left[\frac{F\left(\frac{\sum c_i}{n}\right)10^{-D}}{A}\right]$$

where:

R = the average colony-forming unit count in $\log_{10}$ scale per cm^2 of sampling surface

F = total number of mL of stripping fluid added to the sampling cylinder; in this study, F = 5 mL

$\frac{\sum c_i}{n}$ = average of the duplicate colony counts used for each sample collected

D = dilution factor of the plate counts

A = inside area of the scrub cup in cm^2; in this study, A = 3.46 cm^2.

> **NOTE:** The $\log_{10}$ transformation was performed on these data to convert them to a linear scale. A linear scale, more appropriately a $\log_{10}$ linear scale, was a requirement of the statistical models to be used.

Log_{10} reductions at each recovery interval from each of a subject's forearm were calculated by subtracting $\log_{10}$ recovery values at each exposure time from $\log_{10}$ recovery values of the untreated control arm.

A blocked, one-factor Analysis of Variance (ANOVA) model was used:

$$\hat{y} = Blocks + Sample\ Time + e$$

where:

$\hat{y}$ = Log_{10} Reduction = Untreated Log_{10} Recovery – Treated Log_{10} Recovery

Blocks = Each subject used both configurations.

Sample Time

1, if Immediate

2, if 2 hours

3, if 4 hours

e = Error Term

Model Summary:

s
0.0617799

[1] $F = \frac{Adjusted\ \ Mean\ \ Square\ \ Source}{Adjusted\ \ Mean\ \ Square\ \ Error}$; F is the adjusted mean square values divided by adjusted mean square error. The MS_E is s^2, which is 0.0038, and the standard deviation was $\sqrt{s^2} = s =$ 0.062.

[2] The P or P-value is $P(F \geq x^* | H_0 \ true) < \alpha$. The level of significance is $\alpha = 0.05$. * $x = F$ value calculated.

[3] Significant/Not Significant at $\alpha = 0.05$. If $p \leq 0.05$, the test is significant. If $p > 0.05$, it is not significant.

The model was mixed, because the subjects were selected randomly, and the sample times were pre-determined prior to the study, or fixed effects.

The variances (s^2) were checked for equality by looking for any exceptionally high or low values. If there were none, then the Analysis of Variance (ANOVA) was adequate.

The test hypotheses were:

$$H_0: \ s_1^2 = s_2^2 = s_3^2$$

H_A: They were different.

Using the Levene's Test, the p-value = 0.153. This was not statistically significant, so the H_0 (null hypothesis) was not accepted. Because the 95% confidence interval did not overlap, there is no significance difference between them at $\alpha = 0.05$. However, the individual variances were used to calculate the 95% confidence intervals. The 95% Bonferroni confidence limits, which combine the sample times, are presented on Figure S1.

The 95% confidence interval was applied:

$$\mu = \bar{x} \pm z_{\alpha/2} \frac{s}{\sqrt{n}}$$

The confidence intervals are presented in Figure S2.

Supplementary Tables and Figures

Table S1. Characterization and Stability Study Results for 5% CG-101 Gel, Batch #20H05B1. Tables contain acceptance criteria and results gathered at T=0, 1M, 2M, 6M, and 12M post packaging in glass bottles. All data are represented as means from appearance, CG-101 measurement (%), impurity detection, ethanol measurement (%), pH, viscosity, aerobic plate count, specific pathogens detection, and yeast/mold detection assays.

A) Chemical and Physical Stability of 5% CG-101 Gel, Batch #20H05B1 Packaged into Glass Bottles:

Parameter	Acceptance Criteria	Initial (T=0)	T=1M		T=3M	
			RT	40C/75% RH	RT	40C/75% RH
Appearance	Colorless to light yellow transparent gel	Colorless to light yellow transparent gel	Colorless to light yellow transparent gel	Colorless to light yellow transparent gel	Colorless to light yellow transparent gel	Colorless to light yellow transparent gel
Assay of CG-101, %	4.5 – 5.5% (as a sum of choline and geranic acid assay)	5.4	5.4	5.2	5.1	4.8
Impurities	Total Impurities NMT 5 %	ND*	ND*	ND*	0.71	0.45
Assay of Ethanol, %	63-77%	71.4	71.0	69.2	72.1	70.3
pH	6.5-8.5	7.71	7.72	7.68	7.76	7.81
Viscosity, cP (LVT, 4.6rpm)	1,000-5,000	1,500	1,500	1,000	1,500	1,000
Aerobic Plate Count	NMT 100 cfu/g	<10 cfu/g	<10 cfu/g	<10 cfu/g	<10 cfu/g	<10 cfu/g
Specific Pathogens:<br>*E. coli*<br>*P. aeruginosa*<br>*S. aureus*	Absent /g or ml<br>Absent /g or ml<br>Absent /g or ml	Absent<br>Absent<br>Absent	Absent<br>Absent<br>Absent	Absent<br>Absent<br>Absent	Absent<br>Absent<br>Absent	Absent<br>Absent<br>Absent
Yeast and Mold	NMT 10 cfu/g	<10 cfu/g	<10 cfu/g	<10 cfu/g	<10 cfu/g	<10 cfu/g

*ND – Not detected

B) Physical Parameter and Alcohol's assay of 5%CG-101 Gel, Batch #20H05B1Packaged into the Glass Bottle:

Parameter	Acceptance Criteria	Initial (T=0)	T=6M		T=12M
			RT	40C	RT
Appearance	Colorless to light yellow transparent gel	Colorless to light yellow transparent gel	Colorless to light yellow transparent gel	Colorless to light yellow transparent gel	Colorless to light yellow transparent gel
Assay of Ethanol, %	63-77%	71.4	71.6%	72.1	72.3
pH	6.5-8.5	7.71	7.72	7.68	7.49
Viscosity, cP (LVT, 4.6rpm)	1,000-5,000	1,500	1,500	1,000	1,500

Aerobic Plate Count	NMT 100 cfu/g	<10 cfu/g	<10 cfu/g	<10 cfu/g	<10 cfu/g
Specific Pathogens:<br>E. coli<br>P. aeruginosa<br>S. aureus	Absent /g or ml<br>Absent /g or ml<br>Absent /g or ml	Absent<br>Absent<br>Absent	Absent<br>Absent<br>Absent	Absent<br>Absent<br>Absent	Absent<br>Absent<br>Absent
Yeast and Mold	NMT 10 cfu/g	<10 cfu/g	<10 cfu/g	<10 cfu/g	<10 cfu/g

Table S2: Time Kill Study, Percentage and Log_{10} Reduction for *S. aureus* ATCC 29213 with 1, 2 and 5% CG-101.

	**1% CG-101**			**2% CG-101**			**5% CG-101**		
	**0.5 min**	**1 min**	**5 min**	**0.5 min**	**1 min**	**5 min**	**0.5 min**	**1 min**	**5 min**
% Reduction	99.958%	99.989%	99.999%	99.952%	99.988%	99.999%	99.973%	99.989%	100%
Log_{10} Reduction	3.37	3.94	5.21	3.31	3.90	5.21	3.56	3.94	7.73

Table S3. Log_{10} reduction and percent reduction from control of hCoV229E (ATCC #VR-740) following 15- and 30s by the test product: 5% CG-101 gel

**Dilution (- Log_{10})**	**Virus Control**	**Test**		**NTC**	**NC**	**CTC**	**CC**
		**15 seconds**	**30 seconds**				
							0000
-2	NT	CT	CT	NT	NT	++++	N/A
-3	++++	0000	0000	++++	++++	0000	
-4	++++	0000	0000	++++	++++	0000	
-5	++++	0000	0000	++++	++++	NT	
-6	++++	0000	0000	+++0	++0+	NT	
-7	0000	0000	0000	0000	000+	NT	
$TCID_{50}$ (log_{10})	6.50	$\leq$2.50	$\leq$2.50	6.25	6.50	2.50	
**Log_{10} Reduction**	N/A	**$\geq$4.00**	**$\geq$4.00**	N/A			
**Percent Reduction**		**$\geq$99.99**	**$\geq$99.99**				

+ CPE (cytopathic/cytotoxic effect) present
1 CPE (cytopathic/cytotoxic effect) not detected
CC Cell Control
CTC Cytotoxicity Control
NC Neutralization Control
NTC Neutralizer Toxicity Control
NT Not tested
N/A Not applicable
CT Cytotoxicity

Table S4. Log_{10} reduction and percent reduction from control of hCoV229E (ATCC #VR-740) following 5- and 10min by the test product: aqueous solution of CG-101 (5% w/w)

Dilution (- Log_{10})	Virus Control	Test		NTC	NC	CTC	CC
		5 minutes	10 minutes				
							0000
-2	NT	CT	CT	NT	NT	++++	N/A
-3	++++	0000	0000	++++	++++	0000	
-4	++++	0000	0000	++++	++++	0000	
-5	++++	0000	0000	++++	++++	NT	
-6	++++	0000	0000	+++0	0++0	NT	
-7	0000	0000	0000	0000	0000	NT	
$TCID_{50}$ (log_{10})	6.50	≤2.50	≤2.50	6.25	6.00	2.50	
**Log_{10} Reduction**	N/A	**≥4.00**	**≥4.00**	N/A			
**Percent Reduction**		**≥99.99**	**≥99.99**				

+ CPE (cytopathic/cytotoxic effect) present
1 CPE (cytopathic/cytotoxic effect) not detected
CC Cell Control
CTC Cytotoxicity Control
NC Neutralization Control
NTC Neutralizer Toxicity Control
NT Not tested
N/A Not applicable
CT Cytotoxicity

Table S5. Log_{10} microbial recoveries and Log_{10} microbial reductions from control of *S. aureus* (ATCC #6538), by subject; immediately (30min), 2h, and 4h following application of the test product: 5% CG-101 gel

	Immediately Post-Product Application			2 hours Post-Product Application			4 hours Post-Product Application		
Subject	**Untreated Log_{10} Microbial Recovery**	**Treated Log_{10} Microbial Recovery**	**Log_{10} Reduction**	**Untreated Log_{10} Microbial Recovery**	**Treated Log_{10} Microbial Recovery**	**Log_{10} Reduction**	**Untreated Log_{10} Microbial Recovery**	**Treated Log_{10} Microbial Recovery**	**Log_{10} Reduction**
2	*	*	*	*	*	*	*	*	*
4	6.29	0.86	5.43	6.29	0.86	5.43	6.16	0.86	5.30
13	6.31	0.86	5.45	6.24	0.86	5.38	6.17	0.86	5.31
15	6.26	0.86	5.40	6.18	0.86	5.32	6.03	0.86	5.17
1	6.26	0.86	5.40	6.17	0.86	5.31	6.01	0.86	5.15
6	6.32	0.86	5.47	6.21	0.86	5.35	6.19	0.86	5.33
8	6.35	0.86	5.49	6.28	0.86	5.42	6.23	0.86	5.37
11	6.40	0.86	5.54	6.38	0.86	5.52	6.40	0.86	5.54
12	6.34	0.86	5.48	6.27	0.86	5.41	6.36	0.86	5.50
14	6.20	0.86	5.34	6.19	0.86	5.33	6.29	0.86	5.43
5	6.25	0.86	5.39	6.32	0.86	5.46	6.24	0.86	5.38
10	6.30	0.86	5.44	6.16	0.86	5.30	6.20	0.86	5.34

Note: The lowest detectable limit of the study was 0.86 log_{10} CFU/cm^2 *Data unavailable due to an Adverse Event.

Table S6. Mean Log_{10} microbial recoveries and mean Log_{10} microbial reduction from untreated control of *Staphylococcus aureus* (ATCC #6538), and immediately (30min) of the test product: 5% CG-101 gel

**Sample**	**Sample Size***	**Mean**	**Standard Deviation**
Immediate Untreated Log_{10} Microbial Recovery	11	6.30	0.05
Immediate Treated Log_{10} Microbial Recovery	11	0.86	0.00
Immediate Log_{10} Reduction	11	5.44	0.05

* One Subject's data unavailable due to Adverse Event.

Table S7. Mean Log_{10} microbial recoveries and mean Log_{10} microbial reduction from untreated control of *Staphylococcus aureus* (ATCC #6538), and 2-hours following application and 30-minute air-dry of the test product: 5% CG-101 gel

Sample	Sample Size*	Mean	Standard Deviation
2-hour Untreated Log_{10} Microbial Recovery	11	6.24	0.07
2-hour Treated Log_{10} Microbial Recovery	11	0.86	0.00
2-hour Log_{10} Reduction	11	5.38	0.07

* One Subject's data unavailable due to Adverse Event.

Table S8. Mean Log_{10} microbial recoveries and mean Log_{10} microbial reduction from untreated control of *Staphylococcus aureus* (ATCC #6538), and 4-hours following application and 30-minute air-dry of the test product: 5% CG-101 gel

Sample	Sample Size*	Mean	Standard Deviation
4-hour Untreated Log_{10} Microbial Recovery	11	6.21	0.12
4-hour Treated Log_{10} Microbial Recovery	11	0.86	0.00
4-hour Log_{10} Reduction	11	5.35	0.12

* One Subject's data unavailable due to Adverse Event.

Table S9. Initial and Final Populations of the *Staphylococcus aureus* (ATCC #6538): Inoculum Suspensions

Test Date	Inoculum Tube #	Initial Population (CFU/mL)	Final Population (CFU/mL)
07/06/2020	1	8.45×10^8	9.60×10^8
07/06/2020	2	1.22×10^9	9.90×10^8
07/10/2020	3	1.02×10^9	1.10×10^9

Table S10. General Linear Model: Untreated versus Subject, Sample Time

Factor	Type	Levels	Values
Subject	Random	11	1, 4, 5, 6, 8, 10, 11, 12, 13, 14, 15
Sample Time	Fixed	3	1, 2, 3

Source	DF	Seq SS	Contribution	Adj SS	Adj MS	F-Value[1]	P-Value[2]	Significance[3]
Subject	10	0.14619	54.58%	0.14619	0.014619	3.83	0.005	Significant
Sample Time	2	0.04532	16.92%	0.04532	0.022658	5.94	0.009	Significant
Error	20	0.07634	28.50%	0.07634	0.003817			
Total	32	0.26784	100.00%					

Table S11. Test for Equal Variances: 95% Bonferroni Confidence Intervals for Standard Deviations

Sample Time	*N*	StDev	95% Confidence Interval
Immediate	11	0.054719	(0.0320801, 0.119299)
2-Hour	11	0.070975	(0.0415512, 0.154957)
4-Hour	11	0.119252	(0.0716904, 0.253550)

Table S12. Descriptive Statistics of Log_{10} CFU/cm^2 Reduction Values

Variable	Sample Time	*N*	*N**	Mean	SE Mean	StDev	Minimum	Maximum	Range
Reduction	Immediate (30min)	11	1	5.4392	0.0165	0.0547	5.3424	5.5391	0.1967
	2-Hour	11	1	5.3842	0.0214	0.0710	5.3010	5.5237	0.2227
	4-Hour	11	1	5.3492	0.0360	0.1193	5.1523	5.5391	0.3868

Table S13. Descriptive Statistics Log_{10} CFU/cm^2 Recovery Values

A) Descriptive Statistics: TREATED SITES

Variable	Sample Time	*N*	*N**	Mean	SE Mean	StDev	Minimum	Maximum	Range
Treated	Immediate (30min)	11	1	0.85886	0.000000	0.000000	0.85886	0.85886	0.000000
	2-Hour	11	1	0.85886	0.000000	0.000000	0.85886	0.85886	0.000000
	4-Hour	11	1	0.85886	0.000000	0.000000	0.85886	0.85886	0.000000

B) Descriptive Statistics: UNTREATED SITES

Variable	Sample Time	*N*	*N**	Mean	SE Mean	StDev	Minimum	Maximum	Range
Untreated	Immediate (30min)	11	1	6.2981	0.0165	0.0547	6.2013	6.3979	0.1967
	2-Hour	11	1	6.2431	0.0214	0.0710	6.1599	6.3826	0.2227
	4-Hour	11	1	6.2080	0.0360	0.1193	6.0112	6.3979	0.3868

Figure S1. Test for Equal Variances

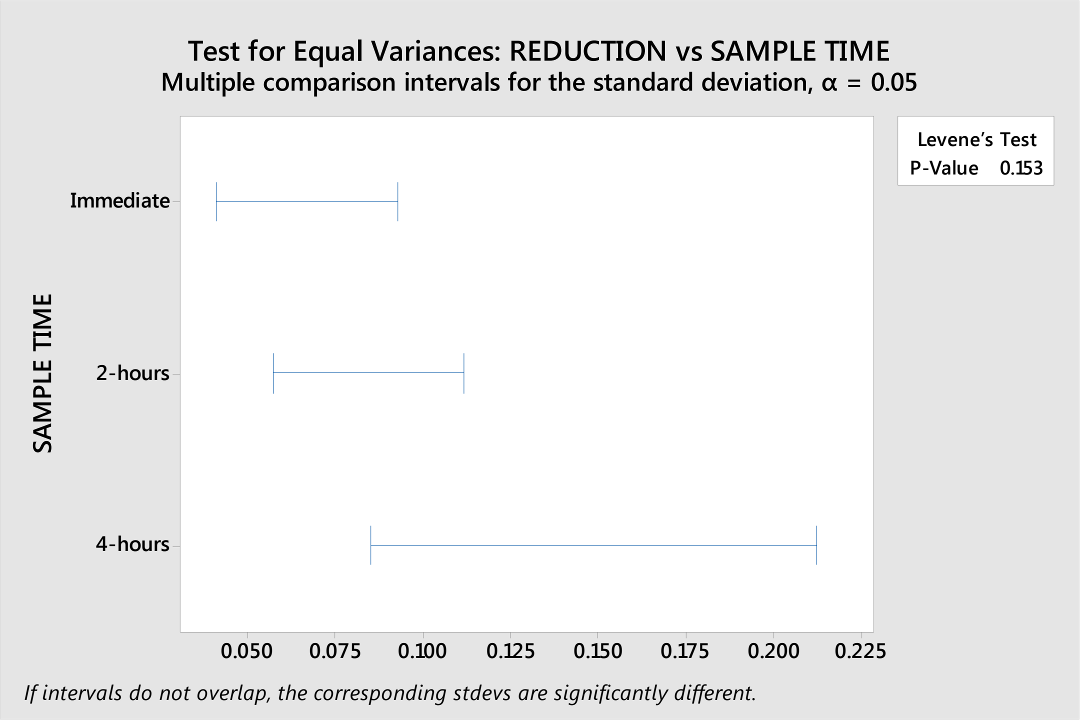

Figure S2. Interval Plot

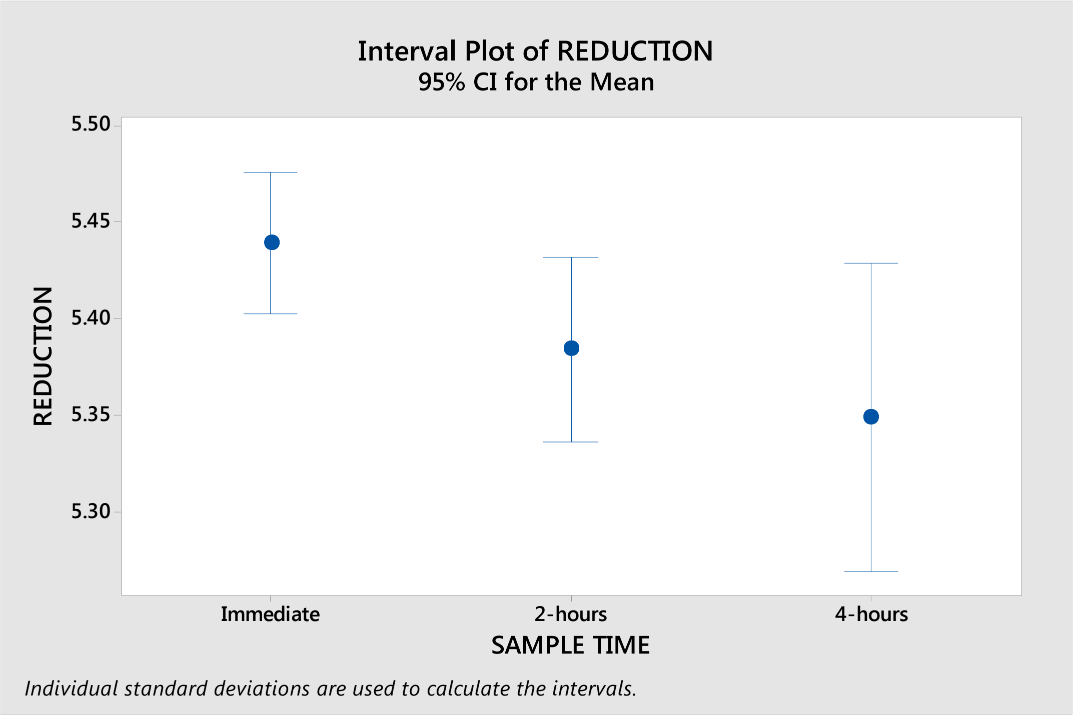

Supplement: Supplementary file 1 — Supporting Information [file GCH2-6-2200064-s001.pdf]
